# Weak Middle-Ear-Muscle Reflex in Humans with Noise-Induced Tinnitus and Normal Hearing May Reflect Cochlear Synaptopathy

**DOI:** 10.1523/ENEURO.0363-17.2017

**Published:** 2017-11-27

**Authors:** Magdalena Wojtczak, Jordan A. Beim, Andrew J. Oxenham

**Affiliations:** Department of Psychology, University of Minnesota, Minneapolis, MN 55455

**Keywords:** cochlear synaptopathy, hidden hearing loss, middle-ear-muscle reflex, noise exposure, stapedial reflex, tinnitus

## Abstract

Chronic tinnitus is a prevalent hearing disorder, and yet no successful treatments or objective diagnostic tests are currently available. The aim of this study was to investigate the relationship between the presence of tinnitus and the strength of the middle-ear-muscle reflex (MEMR) in humans with normal and near-normal hearing. Clicks were used as test stimuli to obtain a wideband measure of the effect of reflex activation on ear-canal sound pressure. The reflex was elicited using a contralateral broadband noise. The results show that the reflex strength is significantly reduced in individuals with noise-induced continuous tinnitus and normal or near-normal audiometric thresholds compared with no-tinnitus controls. Due to a shallower growth of the reflex strength in the tinnitus group, the difference between the two groups increased with increasing elicitor level. No significant difference in the effect of tinnitus on the strength of the middle-ear muscle reflex was found between males and females. The weaker reflex could not be accounted for by differences in audiometric hearing thresholds between the tinnitus and control groups. Similarity between our findings in humans and the findings of a reduced middle-ear muscle reflex in noise-exposed animals suggests that noise-induced tinnitus in individuals with clinically normal hearing may be a consequence of cochlear synaptopathy, a loss of synaptic connections between inner hair cells (IHCs) in the cochlea and auditory-nerve (AN) fibers that has been termed hidden hearing loss.

## Significance Statement

Chronic tinnitus is a prevalent condition that in some cases can lead to debilitating consequences. It may also indicate some damage to the inner ear, termed cochlear synaptopathy, even in cases of clinically normal hearing. However, there are currently no objective diagnostic tests for either tinnitus or cochlear synaptopathy in humans. This study compares the strength of the middle-ear muscle reflex in people with clinically normal or near-normal hearing but suffering from noise-induced tinnitus to that in an age-matched control group without tinnitus. The results show that the tinnitus group had greatly reduced reflex strength. The outcomes are consistent with recent results obtained in mice and suggest that it may be possible to diagnose tinnitus and cochlear synaptopathy in humans.

## Introduction

Tinnitus, the chronic perception of sound in the absence of an acoustic source, affects ∼10-15% of the adult human population worldwide (Eggermont and Roberts, 2015). Most individuals regard tinnitus as a nuisance, but the disorder is considered debilitating in ∼2-4% of the population, causing sleep deprivation, anxiety, and depression, adversely affecting work performance, and resulting in a severe decline in the quality of life (Dobie, 2003; Sanchez, 2004). Despite its public-health relevance, there is currently no objective diagnostic test for tinnitus and no cure (Eggermont, 2015; Seidman and Ahsan, 2015). Tinnitus is typically viewed as resulting from maladaptive neural compensation in response to a deprivation of peripheral input (Noreña and Eggermont, 2003; Schaette and Kempter, 2006; Henry et al., 2014; Schaette, 2014; Eggermont, 2015; Chambers et al., 2016). The fact that many people with clinically normal hearing experience tinnitus (Barnea et al., 1990; Sanchez et al., 2005; Ibraheem and Hassaan, 2017) has been explained in terms of sub-clinical hearing loss or, more recently, in terms of cochlear synaptopathy (Schaette and McAlpine, 2011; Henry et al., 2014). In animals, cochlear synaptopathy has been well documented using postmortem confocal analyses of immunostained tissue that enable counting of presynaptic inner-hair-cell (IHC) ribbons and postsynaptic auditory-nerve (AN) terminals (Kujawa and Liberman, 2009). This assay has revealed a permanent diffuse loss of synaptic IHC/AN connections following just a single exposure to high-level noise, despite no measurable permanent changes to cochlear function or hearing sensitivity (Kujawa and Liberman, 2009; Lin et al., 2011). Because such counts cannot be obtained from live humans, noninvasive physiologic measures, such as auditory brainstem responses (ABRs), have been used in humans reporting excessive noise exposure (Stamper and Johnson, 2015; Liberman et al., 2016) and tinnitus (Schaette and McAlpine, 2011; Guest et al., 2017) to infer the synaptic loss. In animals with noise-induced cochlear synaptopathy, a reduction in amplitude of ABR wave I in response to high-level tones is always observed as a consequence of synaptic loss (Kujawa and Liberman, 2009). In normal-hearing humans with tinnitus, a reduction of the ABR wave I amplitude has not been observed consistently (Gilles et al., 2016; Guest et al., 2017). This is likely because ABR wave I is also dependent on factors unrelated to AN function, such as the skull size and thickness (Eggermont et al., 2007), and interindividual differences in this measure may obscure differences that are due to cochlear synaptopathy. Other measures, such as the electroencephalographic (EEG) envelope following response (EFR), linked to cochlear synaptopathy in mice (Shaheen et al., 2015) and to a reduced ability to process fine-grain temporal information in humans (Bharadwaj et al., 2015), also are not consistently related to noise exposure or tinnitus in humans (Guest et al., 2017), underscoring the need for sensitive noninvasive diagnostic tests for tinnitus and cochlear synaptopathy.

The aim of this study was to investigate the relationship between tinnitus and the strength of the middle-ear-muscle reflex (MEMR) in humans with normal and near-normal audiometric thresholds. A wideband measure of the MEMR was used to probe the reflex strength because this measure is more sensitive to changes in middle-ear impedance than the standard clinical measure, which uses a low-frequency probe tone (Feeney and Keefe, 2001; Schairer et al., 2007; Keefe et al., 2017). There are several reasons why the wideband MEMR measure is expected to be affected by cochlear synaptopathy. First, the MEMR is absent or significantly reduced in individuals with auditory neuropathy (Berlin et al., 2005), a more severe AN disorder. Second, the neural circuit of the MEMR involves afferent neurons with relatively high thresholds and low spontaneous firing rates (Liberman and Kiang, 1984; Kobler et al., 1992), which appear to be preferentially affected by cochlear synaptopathy (Furman et al., 2013). Finally, mice with noise-induced synaptopathy (Valero et al., 2016) have a significantly reduced MEMR strength. To our knowledge, the relationship between tinnitus and a wideband measure of MEMR strength has not been previously investigated in humans.

## Materials and Methods

### Listeners

**Figure 1.**
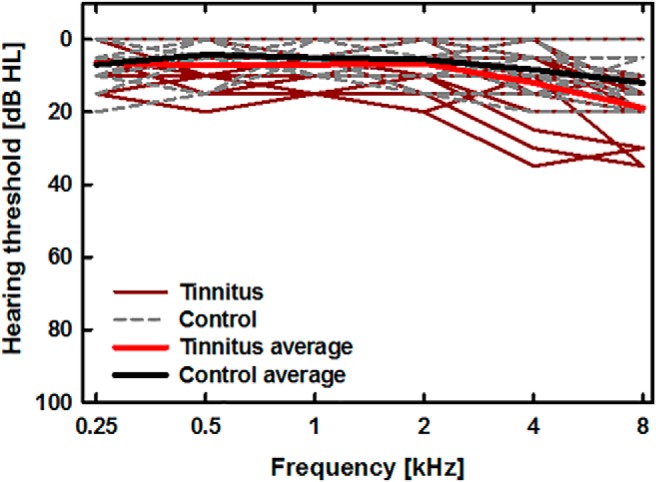
Hearing thresholds for listeners with tinnitus (red solid lines) and without tinnitus (gray dashed lines). The thick light red and black lines show average thresholds for the tinnitus and control groups, respectively. Thresholds were obtained using a calibrated audiometer.

For the study, we recruited 18 individuals with tinnitus (12 male, 6 female) and 18 individuals with no tinnitus (12 male, 6 female). The mean age of the individuals with tinnitus was 46 years (range: 25–63 years) and the mean age of the individuals with no tinnitus was 43 years (range: 27–62 years). Individuals with tinnitus were either employees of the University of Minnesota or were acquaintances of the employees and were recruited because they had complained about experiencing continuous tinnitus. Individuals for the control group were recruited via flyers and personal contacts and were selected to approximately match the ages of the participants in the tinnitus group. Hearing sensitivity was tested using a calibrated audiometer Madsen Conera (GN Otometrics). A method of limits was used to measure hearing thresholds at audiometric frequencies from 250 Hz to 8 kHz in one-octave steps. Each tone was first presented at an audible level between 30 and 45 dB sound pressure level (SPL). The listeners signaled that they heard the tone via a button press. The level of the tone was decreased by 10 dB after each response coinciding with the tone, and increased by 5 dB after the lack of response (the tone not heard). The lowest level that was consistently heard for three repeated tone presentations was taken as hearing threshold. Levels below 0 dB HL were not tested. Figure 1 shows hearing thresholds for all subjects in the control group (gray dashed lines) and the tinnitus group (red solid lines). The thick black and bright red lines represent the mean thresholds for the control and tinnitus group, respectively. Thresholds ≤20 dB HL were considered as indicative of normal hearing sensitivity. Four subjects with tinnitus had mild hearing loss (≤35 dB HL) at high frequencies (≥4 kHz). All the remaining subjects had normal hearing thresholds at all frequencies tested. All subjects reported no history of middle ear disorders, head trauma, or neurologic disorders. Subjects with tinnitus reported that the disorder had been triggered by excessive and repeated noise exposure. Subjects provided informed written consent before participating and the protocol for the study was approved by the Institutional Review Board of the University of Minnesota.

### Stimuli and procedure

The wideband measure of MEMR effects was obtained using clicks with a flat spectrum between 250 Hz and 12 kHz as the probe stimulus and wideband noise bursts as the reflex elicitors. The clicks were generated using a 60th-order recursive exponential filter (Shera and Zweig, 1993). In each trial, clicks were presented for 2 s at a rate of 20 Hz and a level of 90 dB peak-equivalent SPL (peSPL). Because cochlear gain is low for high stimulus levels (Ruggero et al., 1997) and because the rate of click presentation was relatively low, the contribution of medial olivocochlear reflex activation by the click train and by the noise elicitor to changes in ear-canal sound pressure were likely negligible compared with the effects of the MEMR (Veuillet et al., 1991; Guinan et al., 2003). For subjects with unilateral tinnitus (3 out of 18 subjects), the clicks were presented to the ear contralateral to the tinnitus. For subjects with tinnitus that was not lateralized to one side, the clicks were presented to the left ear. During the click train, wideband Gaussian noise (500 Hz to 10 kHz) was presented for 1 s to the contralateral ear to elicit the MEMR. The noise started 500 ms after the onset of the click train and ended 500 ms before its offset which marked the end of a 2-s trial. Thirty trials separated by 2 s of silence were presented in each run. Subjects manually started each run by pressing the spacebar on a computer keyboard placed on their lap to minimize body movements. The level of the noise was increased between the runs but was constant within a run. For each subject, the test started with the lowest noise level used (63 dB SPL) and was increased by 5 dB for each consecutive run up to the maximum level of 88 dB SPL.

During the sound presentation and recording, the subjects were seated comfortably in a reclining chair in a double-walled sound-insulating booth. The subjects were asked to stay relaxed and as still as possible during the recordings. The entire test took ∼12–14 min per subject.

The stimuli were generated on a PC in Matlab (MathWorks) with a LynxTwo-B sound card (Lynx Studio Technology) using a sampling rate of 48 kHz. The stimuli were presented to the listeners using ER10X probes (Etymotic Research) with the ear tips chosen to best fit the subject’s ear. The ear canal sound pressure was recorded from the ear with the clicks via ER10X microphone and was digitized using the LynxTwo-B sound card before being stored for offline analysis.

### Recorded wave form processing

Recorded waveforms were screened visually for artifacts. On the detection of artifacts, a test was repeated, and the clean recordings were analyzed separately for each elicitor level. The artifacts were detected for only two out of 36 subjects and were due to inadvertent removal of the probe from the ear.

To obtain the average baseline (pre-elicitor) and comparison (during-elicitor) click pressure for each elicitor-level condition, the ear-canal sound pressure wave form during nine clicks preceding the onset of the elicitor and nine clicks during the second half of the elicitor was Fourier transformed. The complex-valued frequency-domain representations for the pre-elicitor and during-elicitor clicks were averaged separately across all 30 trials. Only the clicks during the second half of the elicitor were analyzed to allow for sufficient buildup of the MEMR strength (Møller, 1965). The mean real and imaginary parts of each spectral component of the averaged clicks were then used to calculate the magnitudes of the baseline and the comparison ear-canal sound pressures as a function of frequency and the difference between them was related to the magnitude of the average baseline click pressure at the corresponding frequency. An eight-tap FIR filter (with coefficients of 1/8), implemented using “filtfilt” function in Matlab (MathWorks) to avoid shifts of the resultant pattern, was used to smooth rapid variations in the relative noise-induced changes in click sound pressure across frequency. For each subject, the smoothed absolute values of relative sound pressure changes that exceeded two standard deviations from the mean estimate of the measurement noise were summed for frequencies between 500 Hz and 10 kHz, for each elicitor level. The resultant value was taken as the measure of MEMR strength. The MEMR threshold has often been estimated using measures that are derived from changes in ear-canal impedance, such as admittance, absorbance, or the absorbed power (Feeney and Keefe, 2001; Feeney et al., 2004; Keefe et al., 2017). Our measure of MEMR strength is compatible with these measures when changes due to the medial olivocochlear reflex do not contribute to the noise-induced changes in click pressure.

### Statistical analysis

A repeated-measures ANOVA was performed on the MEMR strength measure with the elicitor level as a within-subjects effect, tinnitus (present or absent) and sex as the between-subjects effects, and age and the average of hearing thresholds at 4 and 8 kHz as covariates. Greenhouse-Geisser corrections were used when Mauchly’s test revealed a violation of sphericity assumption. The effect size for the presence of tinnitus after accounting for effects of age and hearing sensitivity in the ANOVA was assessed by calculating the value of η^2^. In addition, the size of the effect of tinnitus on MEMR strength for the highest elicitor level used (88 dB SPL) was calculated in terms of Cohen’s *d* (Cohen, 1988).

## Results

**Figure 2.**
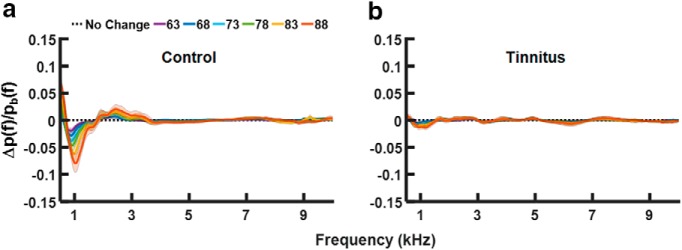
Relative changes in ear-canal sound pressure for clicks due to a contralateral MEMR activator as a function of frequency. ***A***, Data for 18 individuals from the no-tinnitus control group. ***B***, Data from 18 individuals with tinnitus. Different line colors indicate sound pressure changes due to the noise elicitor presented at different levels as shown in the legend in panel ***A***. The shaded areas around the lines represent 95% confidence intervals.

Relative changes in ear-canal sound pressure across frequency elicited by the contralateral noise were averaged separately for the individuals in the control group (Fig. 2*A*) and the group with tinnitus (Fig. 2*B*). Different curves in Figure 2 represent the changes for different elicitor levels, as described in the legend (Fig. 2*A*), and the shaded areas around the curves represent 95% confidence intervals around the means. For individuals in the control group, the pattern of the relative changes in ear-canal sound pressure was similar to the wideband changes in acoustic admittance due to the MEMR reported in previous studies (Feeney and Keefe, 2001; Schairer et al., 2007). The relative change in sound pressure due to the elicitor increased with increasing elicitor level and the maximum negative deflection from zero gradually shifted toward higher frequencies. For individuals with tinnitus the effect of the contralateral noise was greatly reduced (Fig. 2*B*) and there was very little increase of the MEMR effect with increasing elicitor level.

**Figure 3.**
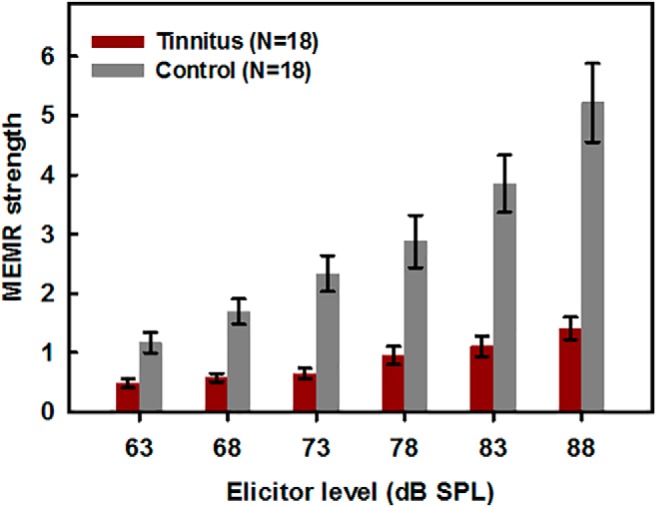
Changes in ear-canal sound pressure for clicks due to a contralateral noise elicitor of the MEMR, summed across frequency. Gray bars show data for the control group without tinnitus, and red bars show data for the group with noise-induced tinnitus.

The values of MEMR strength calculated from the changes in ear-canal sound pressure for each elicitor level, are shown in Figure 3. For all elicitor levels, the reflex strength was greater for the control group (gray bars) than for the tinnitus group (red bars).

**Figure 4.**
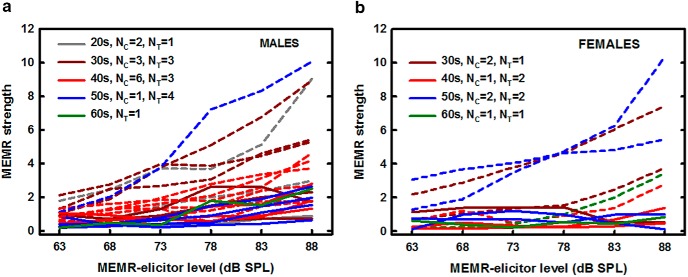
Individual MEMR strength as a function of elicitor level for 24 males (***A***) and 12 females (***B***). Different colors represent data for different age groups depicted by the first number in the legend. Dashed lines show data for individuals from the control group (N_C_) and solid lines show data for individuals with tinnitus (N_T_). Although data for the control group showed much larger variability, no individual with tinnitus exhibited a reflex strength greater than ∼2.5, as estimated by our measure.

Figure 4 shows individual data with different panels showing results for males (Fig. 4*A*) and females (Fig. 4*B*), different colors representing different decades of age, and the dashed and solid lines representing data for individuals in the control and tinnitus groups, respectively. Although a few individuals (males) in the control group had MEMR strength within the range of that for the tinnitus group even for the highest elicitor level used, no individual with tinnitus had a reflex strength (as defined in this study) exceeding a value of ∼2.5.

The ANOVA showed that the effect of age was significant (*F*_(1,30)_ = 2.24, *p* = 0.01), as expected based on existing animal data (Sergeyenko et al., 2013). The effect of the average hearing threshold at the two highest audiometric frequencies (4 and 8 kHz) did not reach significance (*F*_(1,30)_ = 3.38, *p* = 0.08), indicating that elevated thresholds in some of the individuals with tinnitus cannot fully account for the weaker MEMR strength in this study. The effect of sex also was not significant (*F*_(1,30)_ = 0.02, *p* = 0.88) and there was no significant interaction between sex and hearing status (*F*_(1,30)_ = 0.50, *p* = 0.48). Even with the effects of age and high-frequency hearing loss accounted for, there remained a highly significant effect of tinnitus (*F*_(1,30)_ = 31.76, *p* < 0.001, η^2^ = 0.47) indicating significantly weaker MEMR activation in this group of subjects. The within-subjects effect of level was not significant (*F*_(1.41, 42.3)_ = 1.92, *p* = 0.09) but there was a highly significant interaction between level and presence of tinnitus (*F*_(1.41,42.3)_ = 19.45, *p* < 0.001) reflecting the slower growth of the MEMR strength with increasing elicitor level in the tinnitus group. No other interactions were significant.

For the highest level of the elicitor (88 dB SPL) the effect size estimated by a Cohen’s *d* was 3.34, an effect size classified as “huge” (Sawilowsky, 2009), meaning that simply measuring the MEMR at this high sound level can quite reliably differentiate between normal-hearing people with and without tinnitus.

## Discussion

The main finding from this study was a significantly weaker MEMR in humans with tinnitus related to excessive noise exposure but with clinically normal or near-normal hearing than in age-matched humans with similar audiometric hearing but without tinnitus. A recent study in mice found a significantly weaker MEMR in noise-exposed mice with cochlear synaptopathy confirmed by postmortem confocal analysis (Valero et al., 2016). Thus, the MEMR appears to be a robust marker of noise-induced tinnitus in normal- or near-normal-hearing humans, and a robust marker of cochlear synaptopathy in animals. What remains to be determined is a link between tinnitus and cochlear synaptopathy. The difficulty is that in both animals and humans this link can only be inferred, since animals cannot report that they perceive chronic phantom sound and the histopathologic testing of tissue housing IHCs and AN fibers cannot be obtained in live humans.

Nevertheless, there is growing converging evidence from humans and animals suggesting that tinnitus in the absence of audiometric hearing loss is triggered by a diffuse loss of synaptic IHC/AN connections. In mice, eliminating over 95% of synapses with afferent AN fibers via ouabain treatment has been shown to trigger progressive neural gain at the level of the inferior colliculus and the auditory cortex (Chambers et al., 2016) that could result in the perception of phantom sound. This finding is consistent with theoretical models of tinnitus, which postulate that tinnitus results from central neural gain compensating for a loss of peripheral input (Schaette and Kempter, 2006; Parra and Pearlmutter, 2007; Noreña, 2011).

Evidence from humans who have normal hearing but experience chronic tinnitus is based mainly on ABRs which show a reduction in the amplitude of wave I representing the integrity of AN responses in the absence of changes to the amplitude of wave V originating from the brainstem (Schaette and McAlpine, 2011). Small increases in amplitude of ABR wave V were also reported for tinnitus sufferers with mild hearing impairment (Gu et al., 2012) supporting the idea of an increased neural gain in the brainstem structures. Such a relationship was questioned in a recent study by Guest et al. (2017), which did not find a significant reduction in ratio of ABR wave I to wave V amplitudes in young normal-hearing individuals with tinnitus compared to those without tinnitus. In that study, no significant correlation was found between the estimated lifetime noise exposure and the amplitude of ABR wave I or the ratio of wave I to wave V amplitudes. Because tinnitus and noise exposure were significantly correlated, it is possible that the ABR-based measures are simply not sensitive enough to detect cochlear synaptopathy in humans as consistently as in small animals (Kujawa and Liberman, 2009; Lin et al., 2011; Valero et al., 2016).

Given the large size of the effect of tinnitus on MEMR strength after accounting for age and differences in hearing sensitivity at 4 and 8 kHz, and the likely relationship between tinnitus and cochlear synaptopathy, the wideband MEMR measure appears to be a highly promising test for detecting synaptopathy in humans. As shown by individual data in Figure 4, using the wideband MEMR strength as the sole diagnostic test with a criterion value dividing the tinnitus and no-tinnitus individuals set to 2.5, all individuals with tinnitus would be correctly identified as having the phantom sound. The test is not free of false alarms, as four out of 18 individuals (22%) who do not experience tinnitus had MEMR strength below 2.5 for elicitor levels up to 88 dB SPL. It is not clear what accounts for the false positives. One explanation is that the individuals without tinnitus and a weak MEMR may suffer from cochlear synaptopathy but to a degree that does not elicit tinnitus. If that were the case, the MEMR would be a reliable test for diagnosing cochlear synaptopathy. This interpretation is plausible because not all people with hearing impairment, even those with severe hearing loss, experience tinnitus (Eggermont, 2015), indicating that the lack of peripheral input alone does not necessarily result in tinnitus. It has been shown that nonauditory brain areas, such as amygdala in the limbic system and areas of prefrontal cortex, exhibit changes in the presence of tinnitus (Wallhäusser-Franke et al., 2003; Roberts et al., 2013). These findings suggest that although tinnitus is triggered by the lack of peripheral input, central nonauditory areas for processing attention and emotion are important contributors to the perception of phantom sound.

In this study, the test population was limited to individuals with normal and near-normal hearing because of the possible complex relationship between hearing loss and the MEMR. Even for carefully matched audiograms, hearing loss may be due to different combinations of outer-hair cell and IHC damage in different individuals. Disentangling these contributions in humans is time consuming and often unreliable (Johannesen et al., 2014). In mild hearing loss due to outer hair cell damage, cochlear gain is reduced for low intensity sounds but cochlear responses to higher levels that are used to measure the MEMR should be relatively unaffected. IHC damage would reduce the input to the AN and thus could result in an increased MEMR threshold and reduced MEMR strength for all elicitor levels. Previous studies showed little effect of mild to moderate hearing loss on MEMR threshold (Margolis, 1993), but to our knowledge, the effect of hearing loss on the wideband MEMR measure used in this study has not yet been systematically investigated.

We did not measure hearing thresholds for frequencies extending beyond the audiometric range in this study. Elevated hearing thresholds in some of our tinnitus subjects did not account for the sizeable difference in MEMR strength between the two groups. It is possible that the tinnitus group had impaired hearing sensitivity at very high frequencies (>8 kHz) compared to the group without tinnitus. Wideband noises are more effective elicitors of the MEMR that pure tones or narrowband noises (Margolis, 1993), but unfortunately, there appear to be no studies that systematically investigated contributions to the MEMR effects from different frequency regions excited by the elicitor. Popelka et al. (1976) measured MEMR thresholds as a function of increasing frequency band around center frequencies spanning the range from 250 Hz to 4 kHz, but it is not clear from their data whether extending the bandwidth to very high frequencies would affect the strength of the reflex. In fact, for comparable absolute bandwidths (in Hz), MEMR thresholds were higher when measured for noise bands centered at 4 kHz than for noise bands around 1 and 2 kHz. No study has measured the effect of increasing elicitor bandwidth on the wideband MEMR measure, but broadband noise has generally been shown to be a more effective MEMR elicitor than pure tones (Schairer et al., 2007). Thus, the high-frequency contributions to the MEMR have yet to be systematically investigated.

Studies of cochlear synaptopathy in animals have used otoacoustic emissions to evaluate the health of the cochlear responses (Kujawa and Liberman, 2009; Lin et al., 2011). In humans, normal audiometric thresholds are considered to be indicative of normal cochlear function and otoacoustic emissions are not measured in clinical practice except in populations from which reliable behavioral thresholds cannot be obtained, such as newborns, infants, or individuals with severe disabilities. Some studies of cochlear synaptopathy in humans included otoacoustic emissions (Stamper and Johnson, 2015; Prendergast et al., 2016) to investigate correlations of the EEG-based measures with a measure of cochlear function. The clinical measure of MEMR threshold has been shown to be largely unaffected by mild hearing loss (Margolis, 1993), and thus otoacoustic emissions were not measured in this study. However, given a superior sensitivity of the wideband MEMR measure over the current clinical measure, it will be desirable to investigate the correlation between the wideband MEMR strength and the magnitude of otoacoustic emissions in the future. Of course, there may be other disorders associated with a weakened or absent MEMR. For this reason, a history of neurologic disorders would need to be collected as part of the diagnostic battery. In this study, all the subjects reported no such history.

In summary, the MEMR measurement presented here provides a promising objective diagnostic test for tinnitus in humans with no or mild clinical hearing loss. Based on earlier animal studies (Chambers et al., 2016; Valero et al., 2016), we surmise that both the tinnitus in the absence of hearing loss and the reduced MEMR are symptoms of underlying cochlear synaptopathy. Recent neurotrophic treatments have demonstrated some success in restoring lost synaptic connections in animals (Suzuki et al., 2016). For a similar treatment to be viable in humans, an objective but noninvasive test of synaptopathy would be required. The MEMR may provide such a test.

## Synthesis

Reviewing Editor: Leonard Maler, University of Ottawa Decisions are customarily a result of the Reviewing Editor and the peer reviewers coming together and discussing their recommendations until a consensus is reached. When revisions are invited, a fact-based synthesis statement explaining their decision and outlining what is needed to prepare a revision will be listed below. The following reviewer(s) agreed to reveal their identity: Jonathan Siegel.

The revisions were appropriate and the explanation for not including OAE was adequate.

## References

[B1] Barnea G, Attias J, Gold S, Shahar A (1990) Tinnitus with normal hearing sensitivity: extended high-frequency audiometry and auditory-nerve brain-stem-evoked responses. Audiology 29:36–45. 2310352 10.3109/00206099009081644

[B2] Berlin CI, Hood LJ, Morlet T, Wilensky D, St John P, Montgomery E, Thibodaux M (2005) Absent or elevated middle ear muscle reflexes in the presence of normal otoacoustic emissions: a universal finding in 136 cases of auditory neuropathy/dys-synchrony. J Am Acad Audiol 16:546–553. 16295241 10.3766/jaaa.16.8.3

[B50] Bharadwaj HM, Masud S, Mehraei G, Verhulst S, Shinn-Cunningham BG (2015) Individual differences reveal correlates of hidden hearing deficits. J Neuroscience 35:2161–2172. 10.1523/JNEUROSCI.3915-14.2015 25653371 PMC4402332

[B3] Chambers AR, Resnik J, Yuan Y, Whitton JP, Edge AS, Liberman MC, Polley DB (2016) Central gain restores auditory processing following near-complete cochlear denervation. Neuron 89:867–879. 10.1016/j.neuron.2015.12.041 26833137 PMC4760846

[B4] Cohen JI (1988) Statistical power analysis for the behavioral sciences. Mahwah, NJ: Lawrence Erlbaum Associates.

[B5] Dobie RA (2003) Depression and tinnitus. Otolaryngol Clin North Am 36:383–388. 12856305 10.1016/s0030-6665(02)00168-8

[B6] Eggermont JJ (2015) Tinnitus and neural plasticity (tonndorf lecture at xith international tinnitus seminar, berlin, 2014). Hear Res 319:1–11. 10.1016/j.heares.2014.10.002 25316625

[B7] Eggermont JJ, Burkard RF, Don M (2007) Auditory evoked potentials: basic principles and clinical application. Hagerstown, MD: Lippincott Williams & Wilkins.

[B8] Eggermont JJ, Roberts LE (2015) Tinnitus: animal models and findings in humans. Cell Tissue Res 361:311–336. 10.1007/s00441-014-1992-8 25266340 PMC4487353

[B9] Feeney MP, Keefe DH (2001) Estimating the acoustic reflex threshold from wideband measures of reflectance, admittance, and power. Ear Hear 22:316–332. 11527038 10.1097/00003446-200108000-00006

[B10] Feeney MP, Keefe DH, Sanford CA (2004) Wideband reflectance measures of the ipsilateral acoustic stapedius reflex threshold. Ear Hear 25:421–430. 15599190 10.1097/01.aud.0000145110.60657.73

[B11] Furman AC, Kujawa SG, Liberman MC (2013) Noise-induced cochlear neuropathy is selective for fibers with low spontaneous rates. J Neurophysiol 110:577–586. 10.1152/jn.00164.2013 23596328 PMC3742994

[B12] Gilles A, Schlee W, Rabau S, Wouters K, Fransen E, Van de Heyning P (2016) Decreased speech-in-noise understanding in young adults with tinnitus. Front Neurosci 10:288. 10.3389/fnins.2016.00288 27445661 PMC4923253

[B13] Gu JW, Herrmann BS, Levine RA, Melcher JR (2012) Brainstem auditory evoked potentials suggest a role for the ventral cochlear nucleus in tinnitus. J Assoc Res Otolaryngol 13:819–833. 10.1007/s10162-012-0344-1 22869301 PMC3505586

[B14] Guest H, Munro KJ, Prendergast G, Howe S, Plack CJ (2017) Tinnitus with a normal audiogram: relation to noise exposure but no evidence for cochlear synaptopathy. Hear Res 344:265–274. 10.1016/j.heares.2016.12.002 27964937 PMC5256478

[B15] Guinan JJ Jr, Backus BC, Lilaonitkul W, Aharonson V (2003) Medial olivocochlear efferent reflex in humans: otoacoustic emission (oae) measurement issues and the advantages of stimulus frequency OAEs. J Assoc Res Otolaryngol 4:521–540. 10.1007/s10162-002-3037-3 12799992 PMC3202740

[B16] Henry JA, Roberts LE, Caspary DM, Theodoroff SM, Salvi RJ (2014) Underlying mechanisms of tinnitus: review and clinical implications. J Am Acad Audiol 25:5–22. quiz 126. 10.3766/jaaa.25.1.2 24622858 PMC5063499

[B17] Ibraheem OA, Hassaan MR (2017) Psychoacoustic characteristics of tinnitus versus temporal resolution in subjects with normal hearing sensitivity. Int Arch Otorhinolaryngol 21:144–150. 10.1055/s-0036-1583526 28382121 PMC5375708

[B18] Johannesen PT, Pérez-González P, Lopez-Poveda EA (2014) Across-frequency behavioral estimates of the contribution of inner and outer hair cell dysfunction to individualized audiometric loss. Front Neurosci 8:214. 10.3389/fnins.2014.00214 25100940 PMC4108034

[B19] Keefe DH, Feeney MP, Hunter LL, Fitzpatrick DF (2017) Aural acoustic stapedius-muscle reflex threshold procedures to test human infants and adults. J Assoc Res Otolaryngol 18:65–88. 10.1007/s10162-016-0599-z 27957612 PMC5243268

[B20] Kobler JB, Guinan JJ Jr, Vacher SR, Norris BE (1992) Acoustic reflex frequency selectivity in single stapedius motoneurons of the cat. J Neurophysiol 68:807–817. 1432049 10.1152/jn.1992.68.3.807

[B21] Kujawa SG, Liberman MC (2009) Adding insult to injury: cochlear nerve degeneration after “temporary” noise-induced hearing loss. J Neurosci 29:14077–14085. 10.1523/JNEUROSCI.2845-09.2009 19906956 PMC2812055

[B22] Liberman MC, Kiang NY (1984) Single-neuron labeling and chronic cochlear pathology. IV. Stereocilia damage and alterations in rate- and phase-level functions. Hear Res 16:75–90. 6511674 10.1016/0378-5955(84)90026-1

[B23] Liberman MC, Epstein MJ, Cleveland SS, Wang H, Maison SF (2016) Toward a differential diagnosis of hidden hearing loss in humans. PLoS One 11:e0162726. 10.1371/journal.pone.0162726 27618300 PMC5019483

[B24] Lin HW, Furman AC, Kujawa SG, Liberman MC (2011) Primary neural degeneration in the guinea pig cochlea after reversible noise-induced threshold shift. J Assoc Res Otolaryngol 12:605–616. 10.1007/s10162-011-0277-0 21688060 PMC3173555

[B25] Margolis RH (1993) Detection of hearing impairment with the acoustic stapedius reflex. Ear Hear 14:3–10. 8444335 10.1097/00003446-199302000-00002

[B26] Møller AG (1965) An experimental study of the acoustic impedance of the middle ear and its transmission properties. Acta Otolaryngol 60:129–149. 10.3109/00016486509126996 14337949

[B27] Noreña AJ (2011) An integrative model of tinnitus based on a central gain controlling neural sensitivity. Neurosci Biobehav Rev 35:1089–1109. 10.1016/j.neubiorev.2010.11.003 21094182

[B28] Noreña AJ, Eggermont JJ (2003) Changes in spontaneous neural activity immediately after an acoustic trauma: implications for neural correlates of tinnitus. Hear Res 183:137–153. 13679145 10.1016/s0378-5955(03)00225-9

[B29] Parra LC, Pearlmutter BA (2007) Illusory percepts from auditory adaptation. J Acoust Soc Am 121:1632–1641. 17407900 10.1121/1.2431346

[B30] Popelka GR, Margolis RH, Wiley TL (1976) Effect of activating signal bandwidth on acoustic-reflex thresholds. J Acoust Soc Am 59:153–159. 1249314 10.1121/1.380834

[B31] Prendergast G, Guest H, Munro KJ, Kluk K, Leger A, Hall DA, Heinz MG, Plack CJ (2016) Effects of noise exposure on young adults with normal audiograms I: electrophysiology. Hear Res 344:68–81. 27816499 10.1016/j.heares.2016.10.028 PMC5256477

[B32] Roberts LE, Husain FT, Eggermont JJ (2013) Role of attention in the generation and modulation of tinnitus. Neurosci Biobehav Rev 37:1754–1773. 10.1016/j.neubiorev.2013.07.007 23876286

[B33] Ruggero MA, Rich NC, Recio A, Narayan SS, Robles L (1997) Basilar-membrane responses to tones at the base of the chinchilla cochlea. J Acoust Soc Am 101:2151–2163. 9104018 10.1121/1.418265 PMC3578390

[B34] Sanchez L (2004) The epidemiology of tinnitus. Audiol Med 2:8–17. 10.1080/16513860410027781

[B35] Sanchez TG, Medeiros IR, Levy CP, Ramalho Jda R, Bento RF (2005) Tinnitus in normally hearing patients: clinical aspects and repercussions. Braz J Otorhinolaryngol 71:427–431. 10.1016/S1808-8694(15)31194-0 16446955 PMC9441966

[B36] Sawilowsky S (2009) New effect size rules of thumb. J Mod Appl Stat Methods 8:4597–4599. 10.22237/jmasm/1257035100

[B37] Schaette R (2014) Tinnitus in men, mice (as well as other rodents), and machines. Hear Res 311:63–71. 10.1016/j.heares.2013.12.004 24374091

[B38] Schaette R, Kempter R (2006) Development of tinnitus-related neuronal hyperactivity through homeostatic plasticity after hearing loss: a computational model. Eur J Neurosci 23:3124–3138. 10.1111/j.1460-9568.2006.04774.x 16820003

[B39] Schaette R, McAlpine D (2011) Tinnitus with a normal audiogram: physiological evidence for hidden hearing loss and computational model. J Neurosci 31:13452–13457. 10.1523/JNEUROSCI.2156-11.2011 21940438 PMC6623281

[B40] Schairer KS, Ellison JC, Fitzpatrick D, Keefe DH (2007) Wideband ipsilateral measurements of middle-ear muscle reflex thresholds in children and adults. J Acoust Soc Am 121:3607–3616. 10.1121/1.2722213 17552712 PMC2041858

[B41] Seidman MD, Ahsan SF (2015) Current opinion: the management of tinnitus. Curr Opin Otolaryngol Head Neck Surg 23:376–381. 10.1097/MOO.0000000000000186 26204362

[B42] Sergeyenko Y, Lall K, Liberman MC, Kujawa SG (2013) Age-related cochlear synaptopathy: an early-onset contributor to auditory functional decline. J Neurosci 33:13686–13694. 10.1523/JNEUROSCI.1783-13.2013 23966690 PMC3755715

[B43] Shaheen LA, Valero MD, Liberman MC (2015) Towards a diagnosis of cochlear neuropathy with envelope following responses. J Assoc Res Otolaryngol 16:727–745. 10.1007/s10162-015-0539-3 26323349 PMC4636593

[B44] Shera CA, Zweig G (1993) Noninvasive measurement of the cochlear traveling-wave ratio. J Acoust Soc Am 93:3333–3352. 8326061 10.1121/1.405717

[B45] Stamper GC, Johnson TA (2015) Auditory function in normal-hearing, noise-exposed human ears. Ear Hear 36:172–184. 10.1097/AUD.0000000000000228 25350405 PMC4374361

[B46] Suzuki J, Corfas G, Liberman MC (2016) Round-window delivery of neurotrophin 3 regenerates cochlear synapses after acoustic overexposure. Sci Rep 6:24907. 10.1038/srep24907 27108594 PMC4842978

[B47] Valero MD, Hancock KE, Liberman MC (2016) The middle ear muscle reflex in the diagnosis of cochlear neuropathy. Hear Res 332:29–38. 10.1016/j.heares.2015.11.005 26657094 PMC5244259

[B48] Veuillet E, Collet L, Duclaux R (1991) Effect of contralateral acoustic stimulation on active cochlear micromechanical properties in human subjects: dependence on stimulus variables. J Neurophysiol 65:724–735. 2051201 10.1152/jn.1991.65.3.724

[B49] Wallhäusser-Franke E, Mahlke C, Oliva R, Braun S, Wenz G, Langner G (2003) Expression of c-fos in auditory and non-auditory brain regions of the gerbil after manipulations that induce tinnitus. Exp Brain Res 153:649–654. 10.1007/s00221-003-1614-2 14508632

